# A Study on Suitability of EAF Oxidizing Slag in Concrete: An Eco-Friendly and Sustainable Replacement for Natural Coarse Aggregate

**DOI:** 10.1155/2015/972567

**Published:** 2015-09-02

**Authors:** Alan Sekaran, Murthi Palaniswamy, Sivagnanaprakash Balaraju

**Affiliations:** ^1^RVS Technical Campus, Sulur, Coimbatore, Tamil Nadu 641402, India; ^2^Kathir College of engineering and Technology, Coimbatore, Tamil Nadu 641402, India

## Abstract

Environmental and economic factors increasingly encourage higher utility of industrial by-products. The basic objective of this study was to identify alternative source for good quality aggregates which is depleting very fast due to fast pace of construction activities in India. EAF oxidizing slag as a by-product obtained during the process in steel making industry provides great opportunity to utilize it as an alternative to normally available coarse aggregates. The primary aim of this research was to evaluate the physical, mechanical, and durability properties of concrete made with EAF oxidizing slag in addition to supplementary cementing material fly ash. This study presents the experimental investigations carried out on concrete grades of M20 and M30 with three mixes: (i) Mix A, conventional concrete mix with no material substitution, (ii) Mix B, 30% replacement of cement with fly ash, and (iii) Mix C, 30% replacement of cement with fly ash and 50% replacement of coarse aggregate with EAF oxidizing slag. Tests were conducted to determine mechanical and durability properties up to the age of 90 days. The test results concluded that concrete made with EAF oxidizing slag and fly ash (Mix C) had greater strength and durability characteristics when compared to Mix A and Mix B. Based on the overall observations, it could be recommended that EAF oxidizing slag and fly ash could be effectively utilized as coarse aggregate replacement and cement replacement in all concrete applications.

## 1. Introduction

Concrete being the largest man made material used on earth is requiring good quality of aggregates in large volumes. The availability of natural coarse aggregate is depleting day by day due to tremendous demand in Indian infrastructure industry. Aggregates are the main ingredient of concrete occupying 70–80% of its volume and exert a significant influence in concrete properties. A need was felt to identify potential alternative source of coarse aggregate to fulfil the future growth aspiration of Indian infrastructure industry [1].

Use of by-products such as slag, dust, or sludge from the metallurgical industries as filler materials in concrete helps to conserve natural resources as an economically positive option.

Slag, an industrial by-product of steel and iron smelting operations, must be recycled because it has increased proportionately with the development of the steel industry [2, 3].

Big steel plants in India generate about 29 million of tonnes of waste material annually. Slag reduces porosity and permeability of soil, thus increasing the water logging problem. Since large quantities of these wastes are generated daily, they are considered problematic and hazardous for both the factories and the environment. Problem of disposing this slag is very serious which can be reduced by utilizing steel slag for concrete production [4].

Steel making slag specifically generated from EAFs, BOFs, and BFs during the iron and steel making process has many important and environmental uses. In many applications, due to its unique physical structure, slag outperforms the natural aggregate for which it is used as a replacement [5]. Several studies proved that the use of steel slag in concrete as aggregate improves the mechanical and durability properties [6–29].

A very few researches have been performed regarding the utilization of EAF oxidizing slag in concrete. EAF oxidizing slag is an industrial by-product obtained from the steel manufacturing industry. It is produced in large quantities during the steel making operation which utilizes Electric Arc Furnace oxidation process.

EAF oxidizing slag has a high specific gravity; it can produce heavy weight concrete if used as an aggregate for structural concrete. Since most heavy weight aggregates are obtained through quarrying, substitute aggregates must be developed for environmental preservation and protection. From this point of view, the use of EAF oxidizing slag as aggregates holds great significance [30, 31].

According to recent studies, an increase in the compressive strength of concrete was reported if EAF oxidizing slag aggregates are used for structural concrete. The use of EAF oxidizing slag as aggregates for structural concrete not only protects the environment but also reduces costs [32–35]. Kim et al. carried out flexural test on simply supported RC beams to estimate the flexural behaviour of RC beams with EAF oxidizing slag aggregate, and the experimental results were compared with the flexural performance of RC beam with natural aggregates [36].

Kim et al. conducted a research on the characteristics of concrete with EAF oxidizing slag as an aggregate and that evaluated the applicability of the slag for reinforced concrete (RC) members. The study performed bond performance between the steel bar and the concrete with EAF oxidizing slag aggregates which were evaluated in order to use this new material in RC members [37].

Kim et al. conducted results and characteristics of EAF oxidizing slag as an aggregate for structural concrete. The experimental results showed that applying EAF oxidizing slag aggregates to PHC piles enhances the compressive strength, saves energy, lowers carbon dioxide emissions, reduces the amount of cement used, and helps to cut costs [38].

This present paper examines EAF oxidizing slag as coarse aggregates contributing to environment and enhancing greater strength.

Nowadays, most concrete mixture containing supplementary cementing material fly ash to replace certain amount of cement, thus, is reducing the cost of using Portland cement. Fly ash is the most common supplementary cementing material used in concrete. It has been used successfully to replace Portland cement up to 30% by mass, without adversely affecting the strength and durability of concrete [39].

Several laboratory and field investigations involving concrete containing fly ash had reported to exhibit excellent mechanical and durability properties. The pozzolanic reaction of fly ash is a slow process; its contribution towards the strength development occurs only at later ages [39].

The study was conducted to define and analyse the physical, mechanical, and durability properties of eco-friendly concrete made with 50% EAF oxidizing slag aggregate in addition to 30% fly ash (Mix C) compared with conventional concrete mix (Mix A) and concrete with 30% fly ash (Mix B) of two grades M20 and M30.

## 2. Materials

### 2.1. EAFOS Aggregate

**Figure 1:**
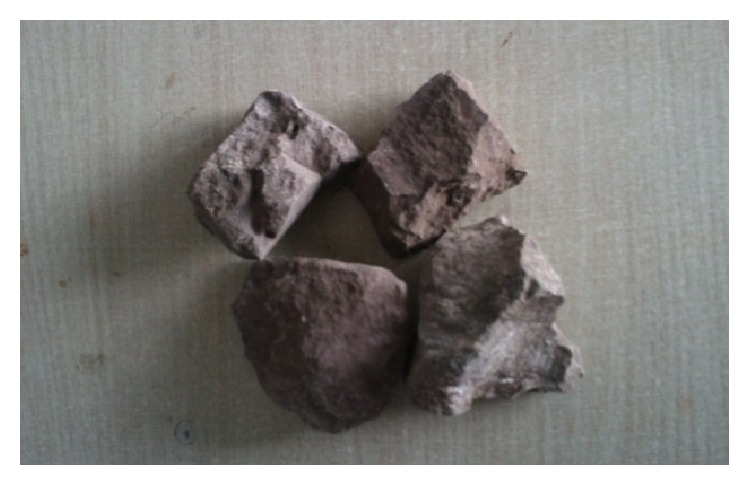
EAF oxidizing slag.

**Figure 2:**
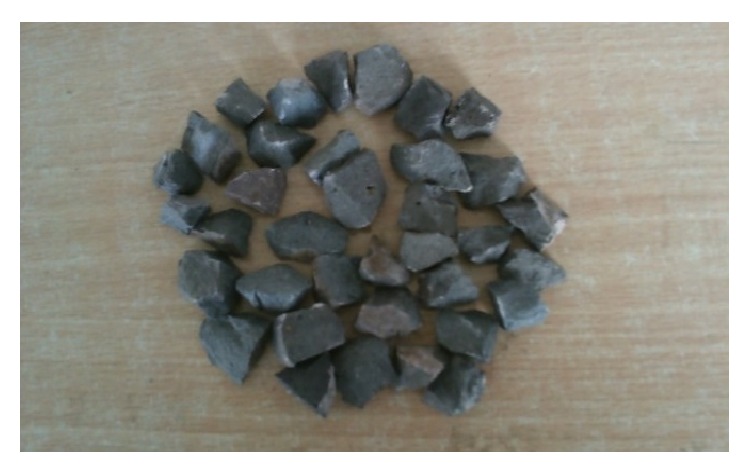
Crushed 20 mm EAF oxidizing slag aggregate.

The slag used in the present investigation was collected from Salem Steel Plant (SSP), Tamil Nadu. The slag had greyish black colour, stone-like appearance, cubical shape, and rough surface texture and more durable than natural aggregate. The roughness and hardness nature of the slag makes it reliable for coarse aggregate. In that study the slag passing through IS Sieve 20 mm was used. EAF oxidizing slag offers high applicability as an aggregate for concrete due to its CaO and SiO_2_ content. The slag is mainly composed of oxides which are similar to the natural rocks and has alkaline properties such as cement products [40]. The physical properties of EAF oxidizing slag are superior to natural coarse aggregate as shown in Table 1. The slag had high density, high alkalinity, higher abrasion resistance, higher crushing strength, and low water absorption. These characteristics give EAF oxidizing slag great potential as an alternative coarse aggregate. The EAF oxidizing slag is shown in Figures 1 and 2.

### 2.2. Fly Ash

In this investigation low calcium fly ash obtained from Mettur Thermal Power Plant was used.

### 2.3. Other Ingredients

53 grade ordinary Portland cement conforming to Indian standard codes IS 12269-1987 was used. Locally available river sand conforming to grading zone II of IS: 383-1970 and crushed granite coarse aggregate of maximum size of 20 mm conforming to IS 383-1970 was used in concrete mixes. Potable water and naphthalene based superplasticizer were used in concrete mixes.

## 3. Mix Proportioning

The mix design of M20 and M30 grade concretes was designed as per IS 10262:2009. The mix proportioning of M20 and M30 grades is shown in Table 2.

## 4. Methodology

### 4.1. Tests on Hardened Concrete

Tests were done as per the following codes of Bureau of Indian Standards. The test for compressive strength on cubes (150 × 150 × 150 mm) was measured at 7, 28, and 90 days of curing as per IS: 516-1959 [41], test for flexural strength on beam (100 × 100 × 500 mm) was measured at 7, 28, and 90 days of curing as per IS: 516-1959 [41], and test for split tensile strength on cylinder (100 × 200 mm) was measured at 7, 28, and 90 days of curing as per IS: 5816-1999 [42].

### 4.2. Coefficient of Water Absorption

Coefficient of water absorption is considered as a measure of permeability of water [43]. This was measured by determining the rate of water uptake by dry concrete in a period of 1 h [44]. The concrete samples were dried at 110°C in an oven for one week until they reached constant weight and then were cooled in a sealed container for one day. The sides of the samples were covered with epoxy resin and were placed partially immersed in water to a depth of 5 mm at one end while the rest of the portions were kept exposed to the laboratory air. The amount of water absorbed during the first 60 min was calculated for Mixes A, B, and C for 28 and 90 days [45]:

(1) $$\begin{array}{c} Ka=\frac{{\left(Q/A\right)}^{2}\cdot 1}{t}, \end{array}$$

where Ka is the coefficient of water absorption (m^2^/s), *Q* is the quantity of water absorbed (m^3^) by the oven-dried specimen in time (*t*), *t* is 3600 s, and *A* is the surface area (m^2^) of concrete specimen through which water penetrates.

### 4.3. Sorptivity

**Figure 3:**
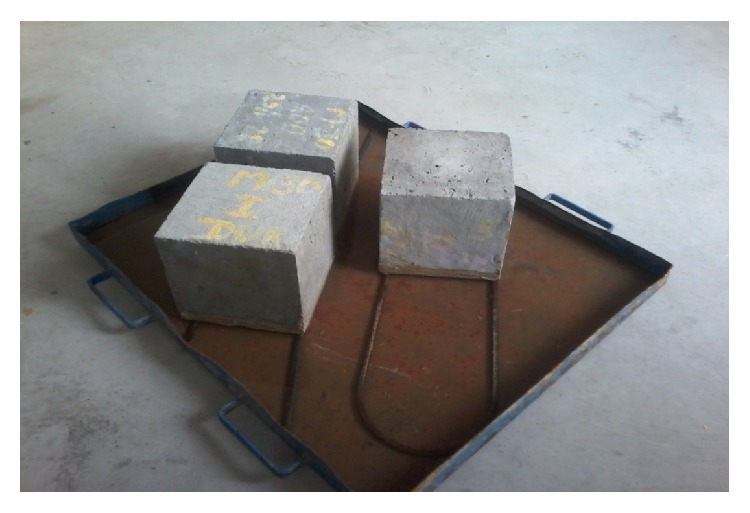
Sorptivity test.

Sorptivity is a measure of the capillary forces exerted by the pore structure causing fluids to be drawn into the body of the material [46]. In this experiment, the speed of water absorption by concrete cubes was considered by measuring the increase in the mass of samples due to water absorption at certain times when only one surface of the specimen is exposed to water. Concrete samples were dried in an oven at 50°C for 3 days and then cooled in a sealed container at 23°C for 15 days as per ASTM C1585 after 28 and 90 days of moist curing [46]. The sides of the concrete samples were covered with epoxy resin in order to allow the flow of water in one direction. The initial mass of the samples was taken after which they were kept partially immersed to a depth of 5 mm in water. The readings were started with initial mass of the sample at selected times after first contact with water (typically 1, 5, 10, 20, 30, 60, 110, and 120 min) [45], the samples were removed, and excess water was blotted off using paper towel and then weighed. Then they were replaced again in water for the chosen time period. The gain in mass per unit area over the density of water was plotted versus the square root of the elapsed time. The slope of the line of best fit of these points was taken as the sorptivity value as per the following equation [47]:

(2) $$\begin{array}{c} i=St^{1/2}, \end{array}$$

where *i* is the cumulative water absorption per unit area of inflow surface (m^3^/m^2^), *S* is the sorptivity (m/s^1/2^), and *t* is the time elapsed (s). The sorptivity test is shown in Figure 3.

### 4.4. Water Penetration

The water penetration test, which is most commonly used to evaluate the permeability of concrete, is the one specified by BS EN-12390-8:2000 [48]. In this test, water was applied on face of the 150 mm concrete cubes specimen under a pressure of 0.5 Mpa. This pressure was maintained constant for a period of 72 hours. After the completion of the test, the specimens were taken out and split open into two halves. The water penetration profile on the concrete surface was then marked and the maximum depth of water penetration in specimens was recorded and considered as an indicator of the water penetration.

### 4.5. Rapid Chloride Permeability Test (RCPT)

**Figure 4:**
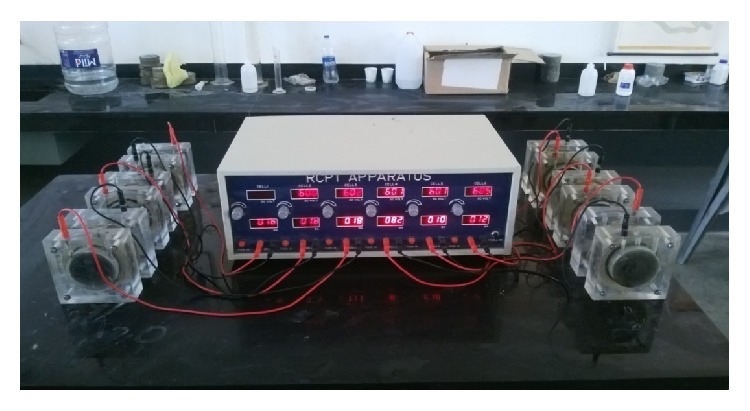
RCPT test.

The resistance of concrete to salt attack was assessed by Rapid Chloride Permeability Test (RCPT) at 28 and 90 days of water curing in conformity with ASTM C-1202 [49]. Three specimens of 100 mm in diameter and 50 mm in thickness which had been conditioned according to the standard were subjected to a 60-V potential for 6 h. The charge pass through the concrete specimens was determined and used to evaluate the chloride permeability of each concrete mixture. The RCPT test is shown in Figure 4.

### 4.6. Alkalinity Test and Resistance to Sulphate Attack

**Figure 5:**
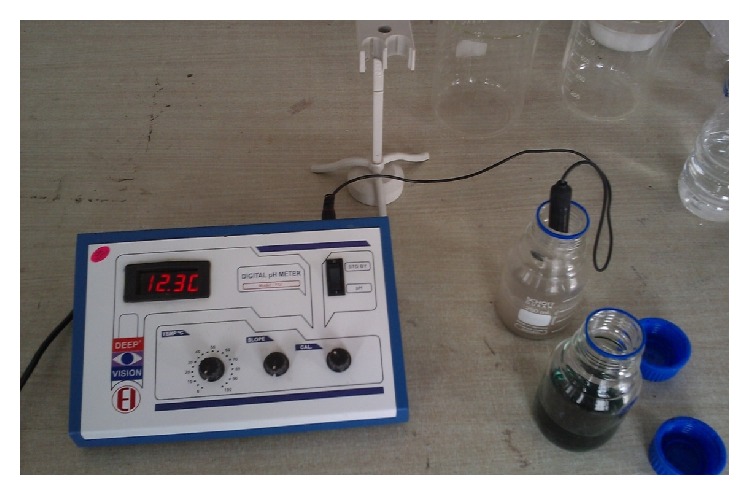
Alkalinity test.

For the alkalinity test, the concrete cubes after curing were dried in an oven for 24 h at 105°C. After cooling in room temperature, the specimens were broken to separate the mortar from the concrete. The mortar is powdered and sieved in 150 *μ*m sieve. 10 g is taken and diluted in distilled water by stirring. The pH value of the solution is noted with a pH meter. The alkalinity test is shown in Figure 5.

The resistance to sulphate attack was studied by immersing the 28 days cured standard cube specimens (150 × 150 × 150 mm) in a solution containing 7.5% magnesium sulphate for 28, 60, and 90 days. The concentration of the solution was maintained throughout the period by changing the solution periodically. The change in weight during the period of 28, 60, and 90 days was determined [50].

## 5. Result and Discussion

**Figure 6:**
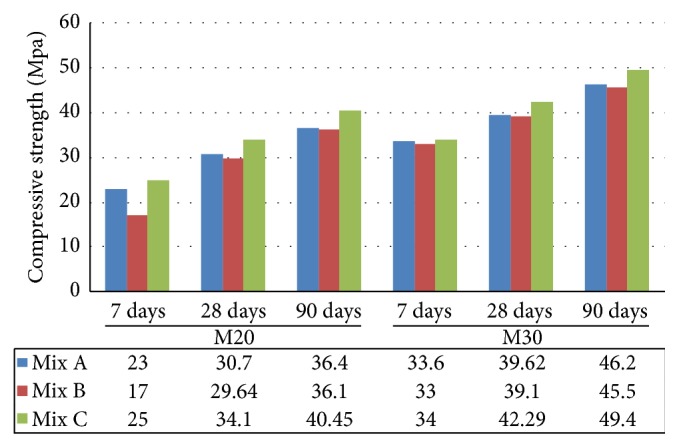
Comparative compressive strength of Mix A, Mix B, and Mix C of M20 and M30 grade.

The compressive test is the most important test that can be used to assure the engineering quality in the application of building material. In M20 grade, at the age of 7 days the compressive strength of Mix C progressively increased by 8.69%  and 47% when compared to Mix A and Mix B, respectively. But the strength development of Mix A increased by 35.29% when compared to Mix B at the earlier age. At the age of 28 and 90 days, there is a continuous improvement in the strength performance of all the concrete mixtures. Mix C increased by 11.07%  and 15.2% at 28 days and 11.11% and 12.04% at 90 days (Figure 6) when compared to Mix A and Mix B. Mix B gained similar strength of Mix A and the strength increased by 52.7% at the later age. Similarly M30 grade of concrete for Mix C progressively increased with all ages of loading and the strength increased by 6.50% and 7.92% at 28 days and 6.9% and 8.5% at 90 days when compared to Mix A and Mix B. From the results, It was observed that the compressive strength of M20 and M30 grade indicated that the higher compressive strength was measured in Mix C (concrete made with EAFOS aggregate and fly ash) in all the ages when compared to Mix A and Mix B.

**Figure 7:**
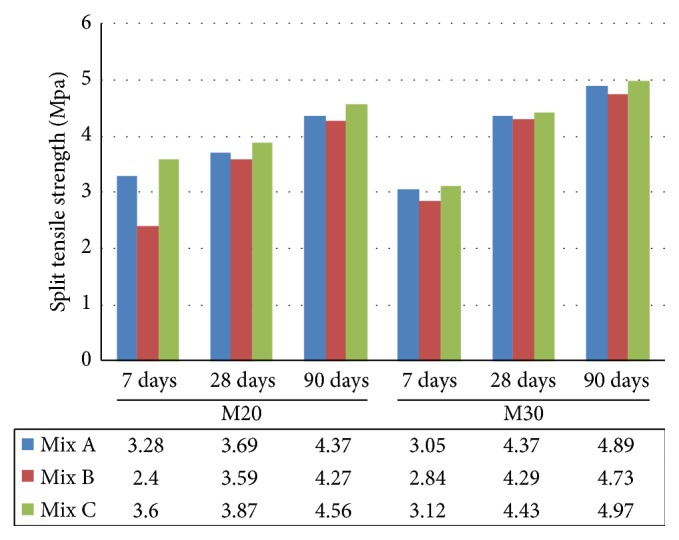
Comparative split tensile strength of Mix A, Mix B, and Mix C of M20 and M30 grade.

The splitting test is well known indirect test for determining the tensile strength of concrete. In M20 grade, at the age of 7 days the split tensile strength of Mix C progressively increased by 9.75%  and 50% when compared to Mix A and Mix B, respectively. But the strength development of Mix A increased by 36.66% when compared to Mix B at the earlier age. At the age of 28 and 90 days (Figure 7), the split tensile strength increased with age in all the concrete mixtures. Mix A and Mix B gained similar strength of Mix C and strength increased by 33.23% and 77.91% at the later age. Similarly M30 grade of concrete for Mix C progressively increased by 2.29% and 9.85% when compared to Mix A and Mix B. But the strength development of Mix A increased by 7.39% when compared to mix B at the earlier days. At the age of 28 and 90 days, the split tensile strength increased with age in all the concrete mixtures. Mix A and Mix B gained similar strength of Mix C and the strength increased by 60.32% and 66.54% at the later age. It was observed that the split tensile strength of M20 and M30 grade indicated that the higher split tensile strength was measured in Mix C at the earlier age and similar strength was measured at the later age.

**Figure 8:**
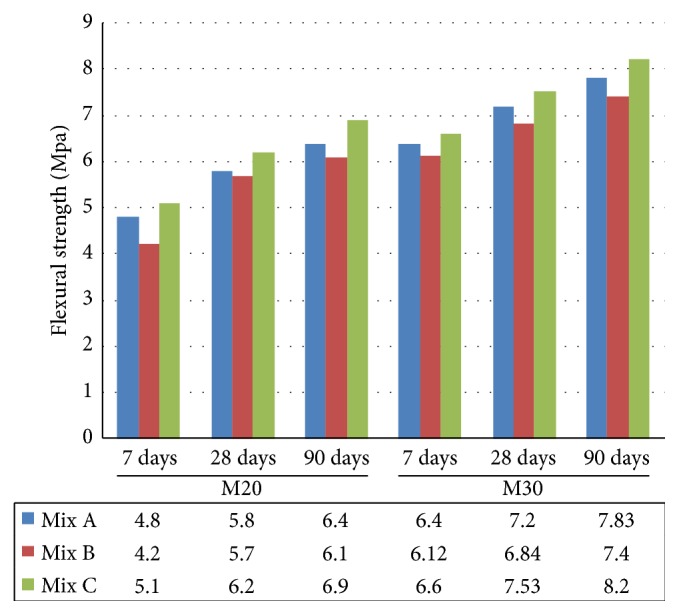
Comparative flexural strength of Mix A, Mix B, and Mix C of M20 and M30 grade.

Flexural test is intended to give the flexural strength of concrete in tension. In M20 grade, at the age of 7 days the flexural strength of Mix C progressively increased by 6.25%  and 21.42% when compared to Mix A and Mix B, respectively. At the age of 28 and 90 days, the flexural strength increased in all the concrete mixtures. Mix C increased by 6.89%  and 8.77% at 28 days and 7.8% and 13.1% at 90 days (Figure 8) when compared to Mix A and Mix B. Similarly M30 grade of concrete for Mix C progressively increased by 3.12% and 7.84% when compared to Mix A and Mix B at the age of 7 days. The strength development of Mix C increased by 4.58% and 10.08% at 28 days and 4.72% and 10.81% at 90 days when compared to Mix A and Mix B. From the results, it was observed that the flexural strength of M20 and M30 grade indicated that the higher flexural strength was measured in Mix C (concrete made with EAFOS aggregate and fly ash) in all the ages when compared to Mix A and Mix B.

**Figure 9:**
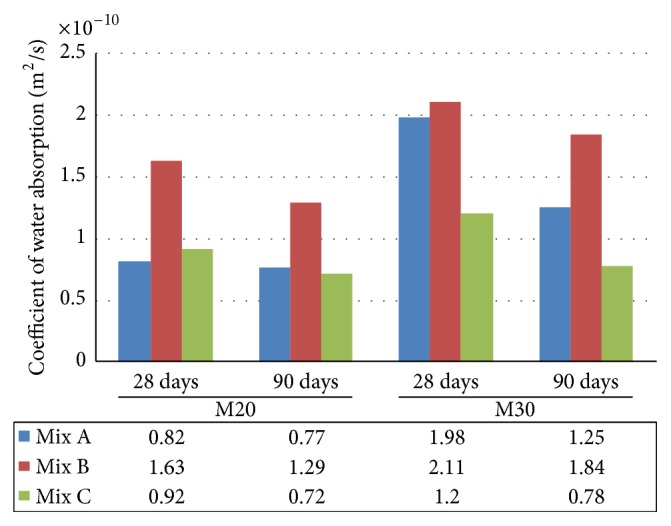
Comparative coefficient of water absorption of Mix A, Mix B, and Mix C of M20 and M30 grade.

The coefficient of water absorption is suggested as a measure of permeability of water. Results indicated that there is significant reduction in the coefficient of water absorption which was measured in Mix C (concrete made with EAF oxidizing slag and fly ash) when compared to Mix A and Mix B for both M20 and M30 grade as shown in Figure 9.

**Figure 10:**
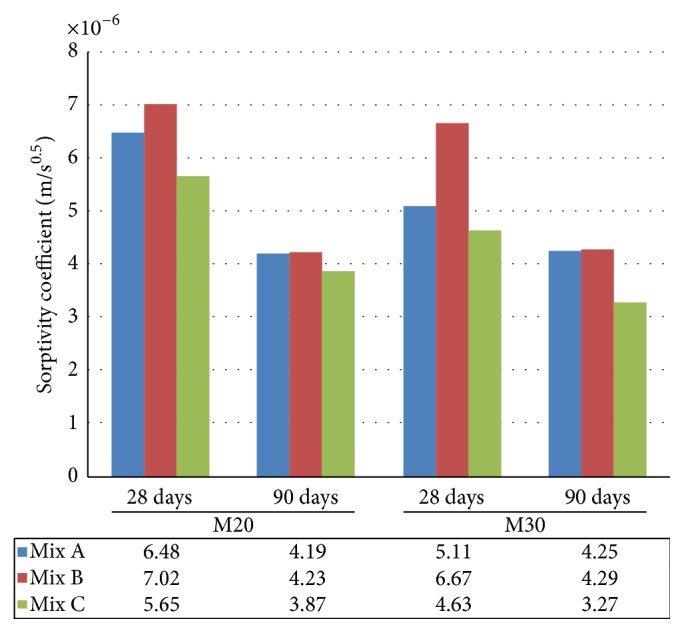
Comparative sorptivity test of Mix A, Mix B, and Mix C of M20 and M30 grade.

The sorptivity test measures capillary suction of concrete when it comes in contact with water. From the results of M20 and M30 grade, at the age of 28 days, Mix C (concrete made with EAFOS aggregate and fly ash) resulted in lesser sorptivity coefficient of 5.65 (10^−6^) (m/s^0.5^) and 4.63 (10^−6^) (m/s^0.5^) when compared to Mix A (conventional concrete) and Mix B (concrete with fly ash), whereas Mix B showed higher sorption and maximum sorptivity coefficient of 7.02 (10^−6^) (m/s^0.5^) and 6.67 (10^−6^) (m/s^0.5^) as compared to Mix A and Mix C. At the age of 90 days (Figure 10), there was further decrease in sorptivity of all the concrete mixes. Mix C showed less sorption (3.87 and 3.27) (10^−6^) (m/s^0.5^) as compared to Mix A and Mix B and the rate of sorptivity of Mix C dropped by 46% and 42%, respectively. The sorptivity coefficient of Mix B was similar to that of Mix A and the rate of sorptivity dropped by 40% and 36% of M20 and M30 grade of concrete, respectively. From the results, it was observed that Mix C (concrete made with EAFOS aggregate and fly ash) obtained lesser sorption in all the ages when compared to Mix A and Mix B of M20 and M30 grade concrete.

**Figure 11:**
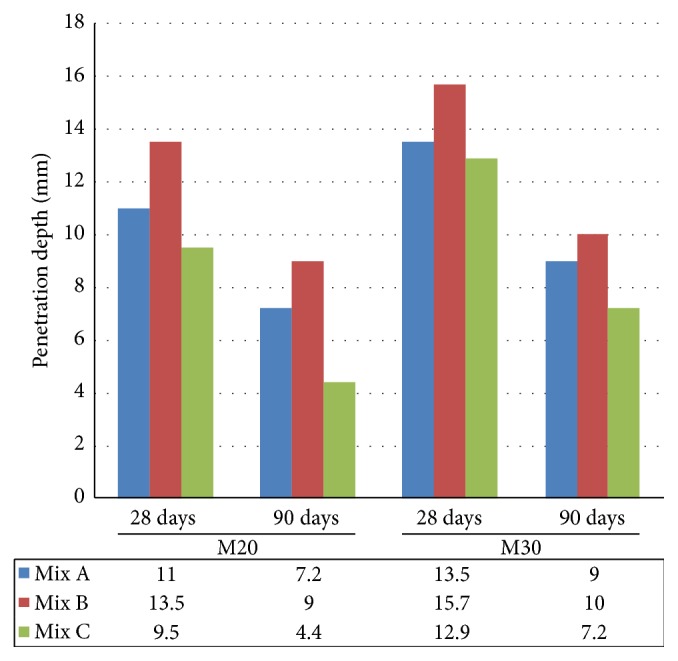
Comparative water penetration test of Mix A, Mix B, and Mix C of M20 and M30 grade.

Water penetration test was used to evaluate the permeability of concrete. From the results, at the age of 28 days, Mix C resulted in lower water penetration depth of 9.5 mm and 12.9 mm as compared to Mix A (11 mm and 13.5 mm) and Mix B (13.5 mm and 15.7 mm) of M20 and M30 grade concrete, respectively. At the curing of 90 days (Figure 11), lower penetration depth was obtained for all concrete mixes and Mix C provided lower water penetration depth of 4.4 mm and 7.2 mm as compared to Mix A and Mix B of M20 and M30 grade concrete, respectively. From the results, it was observed that Mix C, concrete made with EAFOS aggregate and fly ash, would be safe against water permeability.

**Figure 12:**
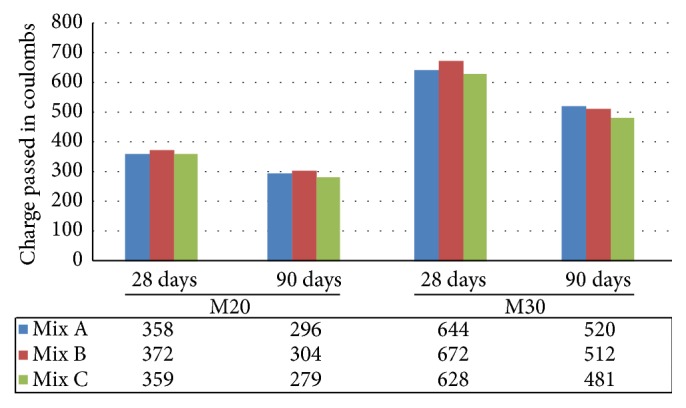
Comparative Rapid Chloride Permeability Test of Mix A, Mix B, and Mix C of M20 and M30 grade.

RCPT is an electrical indication to measure the ability of concrete to resist the penetration of chloride ions. The results of M20 and M30 grade indicated all the values of Mix A, Mix B, and Mix C obtained were between 100 and 1000 and hence the chloride ion permeability is “very low” as per the code. The important observation is that concrete made with EAF oxidizing slag and fly ash (Mix C) makes it less permeable to chloride ions when compared to Mix A and Mix B (Figure 12).

**Figure 13:**
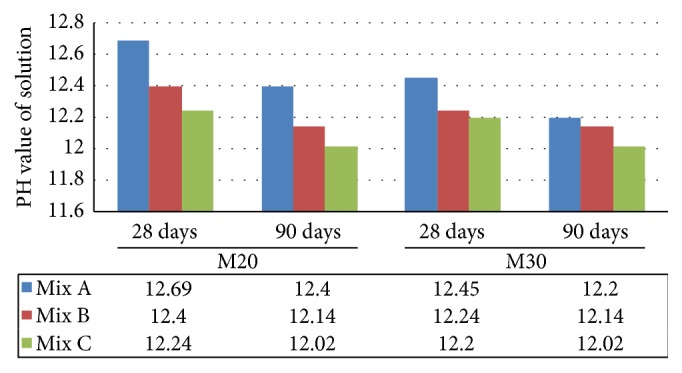
Comparative alkalinity test of Mix A, Mix B, and Mix C of M20 and M30 grade.

**Figure 14:**
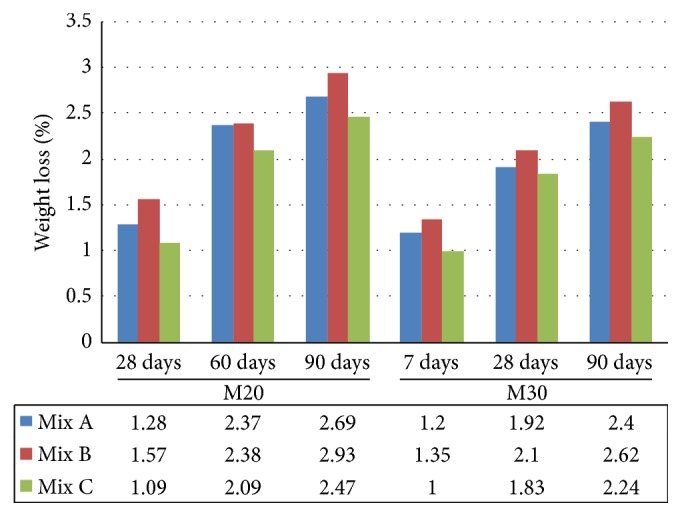
Comparative sulphate attack of Mix A, Mix B, and Mix C of M20 and M30 grade.

Alkalinity test results indicated that the pH value of concrete made with EAF oxidizing slag and fly ash (Mix C) is within the limits about 12 to 13 and hence the potential for corrosion is low, similar to that of Mix A and Mix B (Figure 13). For sulphate attack, it was observed that Mix C got good resistance against sulphate attack when compared to Mix A and Mix B (Figure 14).

## 6. Conclusion

Based on overall results and observations concrete made with EAF oxidizing slag and fly ash exhibited greater strength and durability characteristics compared to conventional concrete mix considered. Thus EAF oxidizing slag, a logical choice for sustaining the environment, eliminates quarrying of natural aggregates and avoids landfill of slags. It could be recommended for all construction activities in India.

## Figures and Tables

**Table 1:** Physical properties of EAF oxidizing slag versus natural coarse aggregate.

Properties	Natural aggregate (IS standard)	Granite aggregate	EAFOS aggregate
Specific gravity	2.6–2.8	2.75	2.9
Bulk density (kg/m^3^)	1.53–1.56	1.53	1.54
Water absorption (%)	1–4	2.5	2
Los Angeles abrasion value (%)	15–20	18.6	16.4
Impact value (%)	Not more than 30% and 40%	28	24.93
Crushing strength (%)	Not more than 30%	26	19.25

**Table 2:** Mix proportioning of M20 and M30 grade.

Grade	Binder				Aggregate				Superplasticizer (kg/m^3^)	w/b	Slump (mm)
	Mix	Fly ash	Cement	Fly ash	Granite	EAFOS	Sand	Water			
	ID	(%)	(kg/m^3^)	(kg/m^3^)	(kg/m^3^)	(kg/m^3^)	(kg/m^3^)	(kg/m^3^)			
M20	A	0	280	0	1250	0	673	142.8	2.8	0.51	80
	B	30	196	84	1250	0	673	142.8	1.96	0.51	95
	C	30	196	84	625	625	673	142.8	1.96	0.51	90

M30	A	0	300	0	1282	0	690	129	3	0.43	90
	B	30	210	90	1282	0	690	129	2.1	0.43	95
	C	30	210	90	641	641	690	129	2.1	0.43	85
